# Prenatal Yoga-Based Interventions May Improve Mental Health during Pregnancy: An Overview of Systematic Reviews with Meta-Analysis

**DOI:** 10.3390/ijerph20021556

**Published:** 2023-01-14

**Authors:** Olga Villar-Alises, Patricia Martinez-Miranda, Javier Martinez-Calderon

**Affiliations:** 1 Departamento de Fisioterapia, Facultad de Enfermería, Fisioterapia y Podología, Universidad de Sevilla, 41009 Sevilla, Spain; 2 Uncertainty, Mindfulness, Self, Spirituality (UMSS) Research Group, 41009 Sevilla, Spain

**Keywords:** anxiety, depression, meta-analysis, pregnancy, prenatal care, systematic review, yoga

## Abstract

An overview of systematic reviews with meta-analysis was developed to summarize evidence on the effectiveness of prenatal yoga-based interventions on pain, psychological symptoms, and quality of life during pregnancy. CINAHL (via EBSCOhost), Embase, PubMed, SPORTDiscus (via EBSCOhost), and the Cochrane Library were searched from inception to 15 December 2022. The intervention of interest was any prenatal yoga-based intervention. Pain, psychological symptoms, and quality of life were considered as outcome measures. The methodological quality of systematic reviews was judged using AMSTAR 2. The primary study overlap among systematic reviews was evaluated, building a citation matrix and calculating the corrected covered area (CCA). A total of ten systematic reviews, including fifteen meta-analyses of interest and comprising 32 distinct primary clinical trials, were included. Meta-analyses on pain and quality of life were not found. Most meta-analyses (93%) showed that prenatal yoga-based interventions are more effective than control interventions in reducing anxiety, depression, and stress symptoms. However, the overall methodological quality of systematic reviews was judged as critically low, and primary study overlap among systematic reviews was very high (CCA = 16%). Altogether, prenatal yoga-based interventions could improve the mental health of pregnant women, although due to the important methodological flaws that were detected, future systematic reviews should improve their methodological quality before drawing firm conclusions on this topic.

## 1. Introduction

Pregnancy is a period where important physiological, social, and emotional changes appear, which can lead future mothers to experience low back pain, pelvic pain, anxiety, depression, post-traumatic stress, and changes in quality of life [1,2]. Psychological disturbances, mainly anxiety and depression, are common in both parents [3]. Concretely, approximately 13% of pregnant women are diagnosed with depression, increasing the percentage up to 22% during the first year of postpartum [4]. Anxiety symptoms are also an important concern, whose prevalence ranges from 18% during the first trimester of pregnancy to 25% in the third [5]. Furthermore, prenatal post-traumatic stress disorders reach 3.3% in community samples, rising to 4.0% during postpartum [6]. Physical activity is a promising approach to improve these psychological symptoms during the prenatal or postnatal period [7,8]. Yoga is a mind–body–spirit practice based mainly, but not exclusively, on breathing exercises, meditation techniques, and physical postures [9]. The interest for yoga-based interventions among pregnant women has exponentially grown, showing interesting results in improving different outcomes during pregnancy (e.g., interpersonal connections, labor pain, or quality of life) [10,11]. Prenatal yoga-based interventions are considered as safe and harmless [11,12], and seem to be more effective than other physical activities such as walking or standard prenatal exercises [11]. Several systematic reviews with meta-analysis have recently explored its effectiveness in altering maladaptive psychological symptoms during pregnancy [13,14,15,16]. In this sense, an overview of systematic reviews can help us to better understand the role that prenatal yoga-based interventions can play in pregnancy outcomes, knowledge of the methodological quality and the possible primary study overlap among systematic reviews covering this topic and detect potential methodological flaws and gaps in knowledge that guide future agenda in this research field. The current overview of systematic reviews with meta-analysis aimed to summarize the effectiveness of prenatal yoga-based interventions on pain, psychological symptoms, and quality of life during pregnancy.

## 2. Materials and Methods

We followed the Preferred Reporting Items for Overviews of Systematic Reviews (PRIO-harms) [17] and the PRISMA for abstract [18]. We used the Open Science Framework to prospectively register our review protocol: https://doi.org/10.17605/OSF.IO/R7MC3. 

### 2.1. Data Sources and Search Strategy

CINAHL (via EBSCOhost), Embase, PubMed, SPORTDiscus (via EBSCOhost), and the Cochrane Library were searched by a reviewer from inception up to 15 December 2022. A PubMed search strategy was developed and adapted to the rest of electronic databases when possible. Search filters for publication type were used. A reviewer manually checked the list of references of those systematic reviews that were included. The complete electronic search and manual strategies were reported in Supplementary file S1.

### 2.2. Eligibility Criteria

The PICOs framework (patient, intervention, comparison, outcome, and study design) was followed to establish the eligibility criteria [19]. We only included systematic reviews with meta-analysis published in peer-review journals written in English or Spanish language. Meta-analyses were only selected if it included at least two clinical trials. Pregnant women were the population of interest. We included any prenatal yoga-based intervention alone or in combination with other approaches (i.e., mindfulness training). No control group restrictions were imposed. Pain, psychological symptoms, and quality of life were the outcomes of interest. We excluded [I] Network meta-analysis when direct estimate comparisons were not provided; [II] systematic reviews where meta-analyses included clinical trials with different populations or interventions that did not satisfy our inclusion criteria; [III] review protocols; and [IV] thesis dissertations and conference proceedings of systematic reviews.

### 2.3. Study Selection

We removed and manually checked duplicates using Mendeley Desktop Citation Management Software v1.19.8 [20]. Subsequently, titles and abstracts were screened. The last screening process was to evaluate the full text when abstracts seemed eligible or when abstracts were unavailable. All the steps were conducted by a reviewer. Consensus was not required.

### 2.4. Methodological Quality Assessment

AMSTAR 2 was independently used by two reviewers to judge the methodological quality of systematic reviews [21]. This tool is composed of 16 items that can be rated as ‘Yes’, ‘Partially yes’, or ‘No’. Seven items (2, 4, 7, 9, 11, 13, 15) have been recommended as critical domains. The overall confidence is classified as high (no weaknesses or one non-critical item), moderate (more than one non-critical item), low (one critical item with or without non-critical items), or critically low (more than one critical item with or without non-critical items). 

### 2.5. Data Extraction and Synthesis

All the results were classified in tables and narratively expressed. A reviewer extracted, from each systematic review, the following information when possible: The first author plus et al.; the year of publication; tools that were used to evaluate methodological quality; the number of clinical trials that were included in our overview; experimental and control group details; outcomes; and main findings. Effect sizes from a subgroup analysis were extracted when the overall effect size was unavailable, or it did not satisfy our eligibility criteria. For this, we prioritized the following order: [I] End time point; [II] experimental group; [III] control group; and [IV] population. We also extracted certainty in the evidence based on the Grading of recommendations assessment, development, and evaluation (GRADE) approach for those systematic reviews that evaluated it. Primary study overlap among systematic reviews was evaluated by a reviewer. A citation matrix was built and the corrected covered area (CCA) was calculated [22]. The CCA represents the area that is covered after removing studies the first time they are counted. Primary study overlap can be classified as slight (CCA 0–5%); moderate (CCA 6–10%); high (CCA 11–15%); or very high (CCA > 15%) [22]. Graphical bibliometric maps were developed by a reviewer using the software VOSviewer 1.6.18 (www.vosviewer.com/, accessed on 15 December 2022). This tool can help us to understand possible interactions between systematic reviews that cover a similar topic using keywords that were reported by each systematic review. The co-occurrence analysis used a full counting method based on these keywords.

## 3. Results

**Figure 1:**
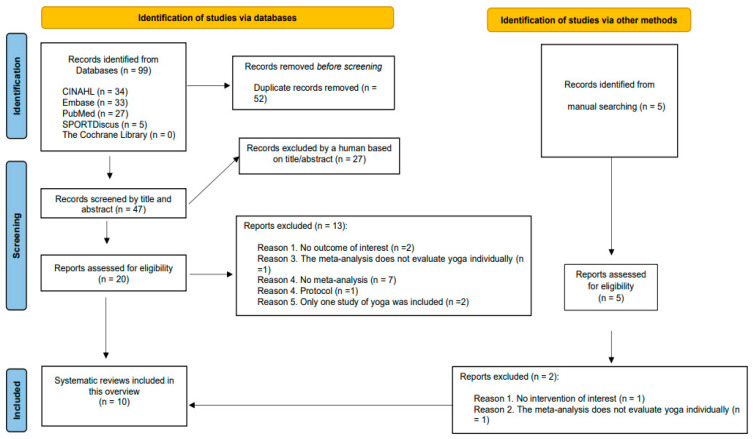
Prisma flow diagram.

We checked a total of 47 titles and abstracts retrieved from electronic databases plus five extra citations that were found after conducting manual searches. Subsequently, 25 full texts were deeply evaluated, and eventually, ten systematic reviews including fifteen meta-analyses and comprising 32 distinct clinical trials were selected [13,14,15,16,23,24,25,26,27,28] (Figure 1). 

A list including full references of those citations that were excluded in the last screening process (n = 15) is reported in Supplementary file S2. Supplementary file S3 shows those meta-analyses from included systematic reviews that were excluded for a specific reason. The characteristics of each systematic review are reported in Table 1.

### 3.1. Co-Occurrence Analysis

**Figure 2:**
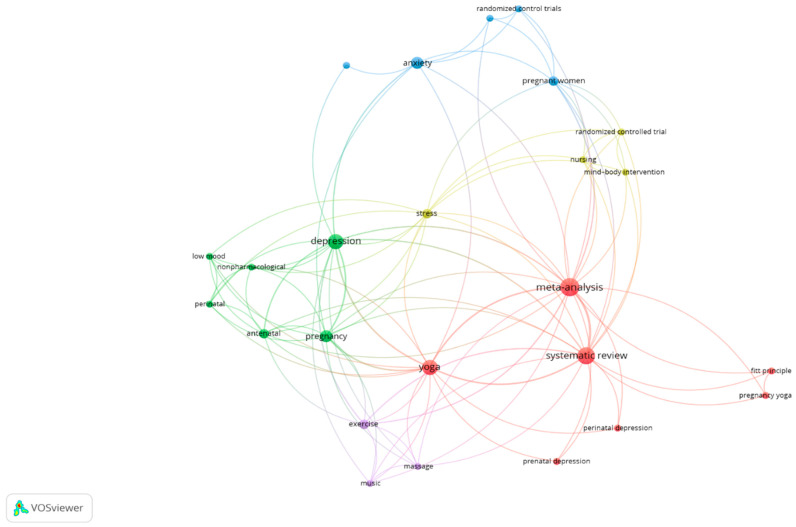
Network visualization analysis.

Figure 2 shows the co-occurrence analysis represented in a network visualization figure. This graphical analysis permits us to observe the interrelation between yoga-based interventions and maladaptive psychological symptoms (e.g., anxiety and depression) using keywords from included systematic reviews. 

### 3.2. Primary Study Overlap among Systematic Reviews

A total of 77 primary studies (double counting) were retrieved from included systematic reviews. Of these, 32 were distinct primary clinical trials. Primary study overlap was evaluated to be very high (CCA = 16%). The citation matrix and the CCA calculation are reported in Supplementary File S4.

### 3.3. AMSTAR 2 Assessment

Table 2 shows AMSTAR 2 judgment item by item for each systematic review. Overall, all systematic reviews were judged to have a critically low quality. The reasons to choose a specific research design (n = 10, 100%), the list of excluded studies (n = 9, 90%), the lack of information to discuss the potential presence of publication bias (n = 9, 90%), and the provision of the sources of funding for those clinical trials that these systematic reviews included (n = 10, 100%) were those AMSTAR 2 items less satisfied. The inter-reviewer agreement was 93.13%. 

### 3.4. Yoga-Based Interventions on Pain and Quality of Life 

No meta-analyses were found for pain or quality of life that satisfied the eligibility criteria.

### 3.5. Yoga-Based Interventions on Anxiety Symptoms 

Prenatal yoga-based interventions were superior no intervention, waitlist, usual care, attention control, information, social support, or active controls to reduce anxiety symptoms, based on six meta-analyses [13,16,24,25,26,27]. 

### 3.6. Yoga-Based Interventions on Depression Symptoms 

Prenatal yoga-based interventions produced more benefits than no intervention, waitlist, usual care, attention control, education, social support, or active controls in decreasing depression symptoms, based on six meta-analyses [13,15,16,23,24,25]. However, prenatal yoga-based interventions were not superior to waitlist, usual care, social support, or education sessions, based on one meta-analysis [28].

### 3.7. Yoga-Based Interventions on Stress Symptoms

Prenatal yoga-based interventions were more effective than no intervention, usual care, or active controls in improving stress symptoms, based on two meta-analyses [13,14]. 

## 4. Discussion

This overview of systematic reviews with meta-analysis aimed to sum up the effectiveness of prenatal yoga-based interventions on pain, psychological symptoms, and quality of life during pregnancy. Surprisingly, no meta-analyses were found exploring the effects of yoga on pain or quality of life. On the other hand, fourteen out of fifteen meta-analyses (93%) underlined the importance of using prenatal yoga-based interventions to reduce anxiety, depression, and stress symptoms. Recent overviews of systematic reviews with meta-analysis in chronic diseases are in line with our results. Specifically, yoga-based interventions may decrease anxiety and depression symptoms in chronic neck pain [29], and improve anxiety, depression, and stress symptoms in different cancer diagnoses [30]. Regular yoga practice may favor a higher interoceptive awareness enhancing insula activity [31]. A greater interoceptive awareness can improve coping strategies to manage stress, whereas major depressive disorders can show a lack of this awareness [32], which may help to better understand the neurobiological effects that yoga can produce to modulate these psychological symptoms and the importance of adhering to a regular yoga practice.

### 4.1. Clinical Implications

Anxiety, depression, and stress symptoms are highly prevalent in pregnant women [6,33]. These psychological factors can alter maternal mental health, which can dramatically affect the child development [34,35,36]. The results in this overview may encourage clinicians to incorporate prenatal yoga-based interventions to manage these maladaptive psychological states. Some pregnant women have narrated that yoga practice helps them to feel more empowered and to develop better coping strategies to overcome fear of childbirth [37,38]. Regular and naïve yoga practitioners should be differed before developing any yoga class. This factor may explain the potential willingness of pregnant women to practice yoga during pregnancy [39]. However, considering the methodological concerns that we will discuss below, we cannot make any strong clinical recommendation. 

### 4.2. Methodological Considerations and Future Agenda

Despite the promising findings of this overview, some methodological concerns raised that should preclude to draw firm conclusions and guide future agenda on this topic. All included systematic reviews were judged to have a critically low quality, based on the use of AMSTAR 2. Many of them did not report details about the development of a review protocol, which is not currently mandatory but highly recommend to foster transparency and a better evidence-based practice [40,41]. Concerns about how methodological steps were conducted in systematic reviews were also alarming. Concretely, the way search strategies were designed, and the lack of transparency during study selection in reporting the reasons to exclude some clinical trials in the last screening process. The lack of information to discuss the potential presence of publication bias was also an important concern. The evaluation of primary study overlap is a novel approach of overview of systematic reviews to detect if we truly need to continue developing new systematic reviews to answer a specific research question with primary available evidence. Our findings invite us to believe that new primary research is needed before conducting new systematic reviews in this field because we observed a very high primary study overlap (CCA = 16%) among systematic reviews. Regarding certainty in the evidence and the extrapolation of prenatal yoga-based interventions into the clinical setting, more than half of systematic review did not judge this certainty and those systematic reviews that used the GRADE approach to do it, found an overall low or very low evidence for outcomes of interest (see Table 1). In addition, no included systematic reviews explored if clinical trials reported sufficient details to replicate their prenatal yoga-based interventions. We recommend using the GRADE approach [42] and the TIDieR checklist [43] respectively, for both purpose. Future agenda should aim to improve the methodological quality of systematic reviews covering this topic, which may follow some recommendations that have been provided in this overview. 

### 4.3. Limitations

We decided to specifically focus on the reported findings by systematic reviews with meta-analysis on prenatal yoga-based interventions. Therefore, systematic reviews covering perinatal or postnatal care were discarded. We also decided to exclusively apply AMSTAR 2 to evaluate the methodological quality of systematic reviews. We are aware that the ROBIS tool could add some extra value regarding the potential risk of bias of systematic reviews [44].

## 5. Conclusions

Most meta-analyses (93%) underlined the importance of practicing prenatal yoga-based interventions in comparison to different controls interventions to improve mental health during pregnancy, concretely, anxiety, depression, and stress symptoms. However, important methodological concerns were observed that preclude to draw firm conclusions about the relevance of prenatal yoga-based interventions to improve mental health in pregnant women. Future systematic reviews with meta-analysis should focus their efforts on exploring other interesting outcomes such as pain or quality of life since no meta-analyses were found for these outcomes and solving the methodological flaws that were detected to improve the quality of systematic review research covering this topic. 

## Figures and Tables

**Table 1:** Characteristics of included systematic reviews.

Author(s) Year	Quality Assessment	Studies Included in This Overview	Interventions	Outcomes	Effect Sizes
Corrigan et al. 2022 [13]	The Cochrane Risk of Bias tool GRADE Available	19	EXPERIMENTAL Yoga CONTROL Usual Care or any active treatment other than yoga	Anxiety: STAI, HADS-A and HAM-A Depression: CES-D, HADS-D, EPDS and HDRS Stress: PSS-10 and PEQ	SMD(95% CI): overall effect ANXIETY SYMPTOMS 11 studies; n = 423 −0.91 (−1.49 to −0.33), *p* = 0.002; I^2^ = 92% DEPRESSION SYMPTOMS 12 studies; n = 679 −0.47 (−0.90 to −0.04), *p* = 0.03; I^2^ = 86% STRESS SYMPTOMS 5 studies; n = 423 −1.22 (−1.66 to −0.79), *p* < 0.00001; I^2^ = 70% * Certainty in the evidence was judged as low for these three outcomes based on the GRADE approach.
Daley et al. 2014 [28]	The scoring system was modified from the Delphi List Criteria GRADE Unavailable	5	EXPERIMENTAL Yoga CONTROL Waitlist, usual care, social support, education sessions	Depression: CES-D and EPDS	SMD(95% CI): subgroup analysis including only depressed women at baseline DEPRESSION SYMPTOMS 5 studies; n = 274 −0.41 (−0.88 to 0.07), *p* = 0.09; I^2^ = 70%
Gong et al. 2015 [23]	Tool for quality assessment Available GRADE Unavailable	6	EXPERIMENTAL Yoga alone/combination CONTROL Usual care or other physical or mental care	Depression: CES-D, HADS and EPDS	SMD (95% CI): Overall effect DEPRESSION SYMPTOMS 6 studies; n = 375 −0.59 (−0.94 to −0.25), *p* = 0.0007; I^2^ = 60%
Guo et al. 2021 [14]	The Cochrane Risk of Bias tool Available GRADE Unavailable	2	EXPERIMENTAL Yoga CONTROL No intervention	Stress: PSS, DASS, PDQ and PSRS	SMD (95% CI): subgroup analysis according to the type of intervention STRESS SYMPTOMS 2 studies; n = 158 −1.07 (−1.55 to −1.59), *p* < 0.0001; I^2^ = 50%
Lin et al. 2022 [24]	Risk of Bias or methodological quality tool Unavailable GRADE Unavailable	13	EXPERIMENTAL Yoga CONTROL Unspecified	Anxiety: STAI, PRAQ-R, SAS, HARS, PASS Depression: CES-D, EPDS, POMS, HDS, HDRS	SMD (95% CI): Overall effect ANXIETY SYMPTOMS 8 studies; n = 516 −0.91 (−1.48 to −0.34), *p* = 0.002; I^2^ = 88% DEPRESSION SYMPTOMS 8 studies; n = 446 −1.58 (−2.31 to −0.85), *p* < 0.0001; I^2^ = 90%
Ng et al. 2019 [15]	The Cochrane Risk of Bias tool Available GRADE Unavailable	6	EXPERIMENTAL Yoga CONTROL No intervention	Depression: EPDS and HADS	SMD (95% CI): overall effect DEPRESSION SYMPTOMS 6 studies; n = unspecified −0.452 (−0.86 to −0.880), *p* = 0.015; I^2^ = 64%
Ningrum et al. 2019 [27]	Risk of Bias or methodological quality tool Unavailable GRADE Unavailable	6	EXPERIMENTAL Yoga CONTROL No intervention, usual care, social support	Anxiety: unspecified	SMD (95% CI): Overall effect ANXIETY SYMPTOMS 6 studies; n = 454 −0.48 (−0.92 to −0.03), *p* = unspecified; I^2^ = 81%
Smith et al. 2019 [16]	The Cochrane Risk of Bias tool GRADE Available	4	EXPERIMENTAL Yoga CONTROL Usual care, waitlist, information, and social support	Anxiety: STAI Depression: EPDS, CES-D and POMS	SMD (95% CI): subgroup analysis according to the type of intervention DEPRESSION SYMPTOMS 4 studies; n = 103 −0.21(−0.48 to −0.06), *p* = 0.13 I^2^ = 0% ANXIETY SYMPTOMS 3 studies; n = 96 −1.60 (−5.67 to −2.46), *p* = 0.44; I^2^ = 54% * Certainty in the evidence was judged as low for these two outcomes based on the GRADE approach.
Wang et al. 2022 [26]	The Cochrane Risk of Bias tool GRADE Unavailable	6	EXPERIMENTAL Yoga CONTROL Usual care, active controls, social support	Anxiety: STAI	SMD (95% CI): overall effect ANXIETY SYMPTOMS 6 studies; n = unspecified −4.75 (−8.3 to −1.19), *p* = unspecified; I^2^ = 73.8%
Zhu et al. 2021 [25]	The Cochrane Risk of Bias tool GRADE Available	10	EXPERIMENTAL Yoga CONTROL Usual care, social support, parenting education, attention control, usual antenatal exercises, perinatal health education	Anxiety: S-STAI, STAI, SAS Depression: EPDS, CES-D, D-HADS	SMD (95% CI): subgroup analysis according to the type of intervention ANXIETY SYMPTOMS 6 studies; n = 393 −0.87 (−1.52 to −0.23), *p* = 0.008; I^2^ = 89% DEPRESSION SYMPTOMS 10 studies; n = 553 −0.45(−0.69 to −0.22), *p* = 0.0002 I^2^ = 43% * Certainty in the evidence was judged as low for depression symptoms and very low for anxiety symptoms based on the GRADE approach.

Note: CES-D: Centre for Epidemiological Studies—Depression; DASS: Depression, Anxiety and Stress Scale; EPDS: Edinburgh Postnatal Depression Scale; GRADE: Grading of recommendations assessment, development and evaluation; HADS: Hospital Anxiety and Depression Scale—Depression; HAM-A: Hamilton Anxiety Rating Scale; HDRS: Hamilton Depression Rating Scale; PASS: Perinatal Anxiety Screening Scale; PEQ: Pregnancy Experiences Questionnaire; POMS: Profile of Mood States; PRAQ-R: Pregnancy-related Anxiety Inventory; PSRS: Pregnancy Stress Rating Scale; PSS: Perceived Stress Scale; SAS: Self-rating Anxiety Scale; SMD: Standard mean difference; STAI: State-Trait Anxiety Inventory; WHOQoL-100: World Health Organization Quality of Life Assessment Instrument.

**Table 2:** AMSTAR 2 judgment.

Review	1	2	3	4	5	6	7	8	9	10	11	12	13	14	15	16	Total
Corrigan et al., 2022 [13]																	CLQR
Daley et al., 2014 [28]																	CLQR
Gong et al., 2015 [23]																	CLQR
Guo et al., 2020 [14]																	CLQR
Lin et al., 2022 [24]																	CLQR
Ng et al., 2019 [15]																	CLQR
Ningrum et al., 2020 [27]																	CLQR
Smith et al., 2019 [16]																	CLQR
Wang et al., 2022 [26]																	CLQR
Zhu et al., 2021 [25]																	CLQR

Note: Answers: Red color: No; yellow color: Partially yes; green color: Yes. Overall Score: CLQR: Critically Low. Quality Review LQR: Low-Quality Review. Items: **AMSTAR 1**: Did the research questions and inclusion criteria for the review include the components of PICO? **AMSTAR 2**: Did the report of the review contain an explicit statement that the review methods were established prior to the conduct of the review and did the report justify any significant deviations from the protocol? **AMSTAR 3**: Did the review authors explain their selection of the study designs for inclusion in the review? **AMSTAR 4**: Did the review authors use a comprehensive literature search strategy? **AMSTAR 5**: Did the review authors perform study selection in duplicate? **AMSTAR 6**: Did the review authors perform data extraction in duplicate? **AMSTAR 7**: Did the review authors provide a list of excluded studies and justify the exclusions? **AMSTAR 8**: Did the review authors describe the included studies in adequate detail? **AMSTAR 9**: Did the review authors use a satisfactory technique for assessing the risk of bias in individual studies that were included in the review? **AMSTAR 10**: Did the review authors report on the sources of funding for the studies included in the review? **AMSTAR 11**: If a meta-analysis was performed, did the review authors use appropriate methods for statistical combination of results? **AMSTAR 12**: If a meta-analysis was performed, did the review authors assess the potential impact of the risk of bias in individual studies on the results of the meta-analysis or other evidence syntheses? **AMSTAR 13**: Did the review authors account for the risk of bias in individual studies when interpreting/discussing the results of the review? **AMSTAR 14**: Did the review authors provide a satisfactory explanation for, and discussion of any heterogeneity observed in the results of the review? **AMSTAR 15**: If they performed a quantitative synthesis, did the review authors carry out an adequate investigation of publication bias (small-study bias) and discuss its likely impact on the results of the review? **AMSTAR 16**: Did the review authors report any potential sources of conflict of interest, including any funding they received for conducting the review?

## Data Availability

Not applicable.

## Supplementary Materials

The following supporting information can be downloaded at: https://www.mdpi.com/article/10.3390/ijerph20021556/s1, Supplementary file S1. Search Strategies in electronic databases and manual searches. Supplementary file S2. Excluded citations in the last screening (n = 15): reasons. Supplementary file S3. Meta-analyses that were excluded from included systematic reviews. Supplementary file S4. Citation matrix and corrected covered area (CCA) calculation.
