# Supplementary material for: Prenatal Yoga-Based Interventions May Improve Mental Health during Pregnancy: An Overview of Systematic Reviews with Meta-Analysis
Source: Int J Environ Res Public Health. 2023 Jan 14;20(2):1556. doi: 10.3390/ijerph20021556 (PMC9863076; doi:10.3390/ijerph20021556)
Supplement: Supplementary file 1 [file ijerph-20-01556-s001.zip › ijerph-2136506-supplementary.pdf]

International Journal of
Environmental Research and Public Health

MDPI

Supplementary file S1. Search Strategies in electronic databases and manual searches.

CINAHL (date 15/12/2022)

TX (yoga OR yogic OR pranayama) AND TI (pregnancy OR pregnant OR prenatal OR antenatal) AND TI (systematic-review OR meta*)

(Studies retrieved = 34)

Search modes - Boolean/Phrase.

Search filter: source: academic publications.

Embase (date 15/12/2022)

(yoga OR yogic OR pranayama) AND ('pregnancy':ti OR 'pregnant':ti OR 'prenatal':ti OR 'antenatal':ti) AND ('systematic review':ti OR 'meta*':ti)

(Studies retrieved = 33)

Search filter: publication type (review OR article OR article in press).

PubMed (date 15/12/2022)

(yoga [all] OR yogic [all] OR pranayama [all]) AND (pregnancy [title] OR pregnant* [title] OR prenatal [title] OR antenatal [title]) AND (systematic review [title] OR meta* [title])

(Studies retrieved= 27)

SPORTDiscus (date 15/12/2022)

TX (yoga OR yogic OR pranayama) AND TI (pregnancy OR pregnant OR prenatal OR antenatal) AND TI (systematic-review OR meta*)

(Studies retrieved = 5)

Search modes - Boolean/Phrase.

Search filter: source: academic publications.

The Cochrane Library (date 15/12/2022)

ID	Búsqueda
#1	(yoga)
#2	(yogic)
#3	(pranayama)
#4	(pregnancy):ti
#5	(pregnant):ti
#6	(antenatal):ti
#7	(prenatal):ti
#8	(systematic review):ti
#9	(meta*):ti
#10	((#1 OR #2 OR #3) AND (#4 OR #5 OR #6 OR #7) AND (#8 OR #9))

(Studies retrieved = 0)

Filter: Cochrane reviews.

Manual searches

Guo et al. 2020:

- Dhillon A, Sparkes E, Duarte R. Mindfulness-based interventions during pregnancy: a systematic review and meta-analysis. Mindfulness. 2017; 8(6):1421–143.
- Excluded. Yoga-based interventions were not separately meta-analyzed.

Lin et al. 2022:

- Ningrum, S.A.; Budihastuti, U.R.; Prasetya, H. Efficacy of yoga exercise to reduce anxiety in pregnancy: A meta-analysis using randomized controlled trials. J. Matern. Child Health 2020, 5, 629–640.
- Included.

  Ningrum et al. 2020:
- Daley AJ, Foster L, Long G, Palmer C, Robinson O, Walmsley H, Ward R. The effectiveness of exercise for the prevention and treatment of antenatal depression: systematic review with meta-analysis. BJOG. 2015 Jan;122(1):57–62.
- Included.

  Daley et al. 2015:
- Rethorst CD, Wipfli BM, Landers DM. The antidepressive effects of exercise: a meta-analysis of randomized trials. Sports Med. 2009;39(6):491–511.
- Excluded. No intervention of interest.

Manual searches including the term perinatal [title] in PubMed.

Although perinatal care was not our objective, we have detected that a systematic review that was based on perinatal care, included a meta-analysis where all clinical trials satisfied our inclusion criteria. Therefore, we decided to include this systematic review in our overview.

Wang G, Liang C, Sun G. Yoga's Therapeutic Effect on Perinatal Depression: A Systematic Review and Meta-Analysis. Psychiatr Danub. 2022 Summer;34(2):195–204.

Supplementary file S2. Excluded citations in the last screening (n = 15): reasons.

	Excluded Reviews	Reason
1.	Adeli Gargari M, Esmailpour K, Mirghafourvand M, Nourizadeh R, Mehrabi E. Effects of Psycho-education Interventions on Perceived Childbirth Fear and Anxiety by Pregnant Women: A Systematic Review and Meta-analysis. International Journal of Women's Health and Reproduction Sciences. 2020;9(4):230–237.	No outcome of interest.
2.	Curtis K, Weinrib A, Katz J. Systematic review of yoga for pregnant women: current status and future directions. Evid Based Complement Alternat Med. 2012;2012:715942.	No meta-analysis.
3.	Dhillon A, Sparkes E, Duarte R. Mindfulness-based interventions during pregnancy: a systematic review and meta-analysis. Mindfulness. 2017;8(6):1421−1143. **(Manual searches)**	The meta-analysis does not evaluate yoga individually.
4.	Domínguez-Solís E, Lima-Serrano M, Lima-Rodríguez JS. Non-pharmacological interventions to reduce anxiety in pregnancy, labour and postpartum: A systematic review. Midwifery. 2021 Nov;102:103126.	No meta-analysis.
5.	Hu X, Ma M, Zhao X, Sun W, Liu Y, Zheng Z, Xu L. Effects of exercise therapy for pregnancy-related low back pain and pelvic pain: A protocol for systematic review and meta-analysis. Medicine (Baltimore). 2020 Jan;99(3):e17318.	Protocol.
6.	Koukoulithras I Sr, Stamouli A, Kolokotsios S, Plexousakis M Sr, Mavrogiannopoulou C. The Effectiveness of Non-Pharmaceutical Interventions Upon Pregnancy-Related Low Back Pain: A Systematic Review and Meta-Analysis. Cureus. 2021 Jan 30;13(1):e13011.	Only one study of yoga was included.
7.	Liu N, Gou WH, Wang J, Chen DD, Sun WJ, Guo PP, Zhang XH, Zhang W. Effects of exercise on pregnant women's quality of life: A systematic review. Eur J Obstet Gynecol Reprod Biol. 2019 Nov;242:170−177.	No meta-analysis.
8.	Lučovnik M, Pravst T, Dinevski IV, Žebeljan I, Dinevski D. Yoga during pregnancy: a systematic review. 2021; 90(3-4):150−158.	No meta-analysis.

	Excluded Reviews	Reason
9.	Matvienko-Sikar K, Lee L, Murphy G, Murphy L. The effects of mindfulness interventions on prenatal well-being: A systematic review. Psychol Health. 2016 Dec;31(12):1415–1434.	No meta-analysis.
10.	Rethorst CD, Wipfli BM, Landers DM. The antidepressive effects of exercise: a meta-analysis of randomized trials. Sports Med. 2009;39(6):491–511.	No intervention of interest.
	**(Manual searches)**	
11.	Rong L, Dai LJ, Ouyang YQ. The effectiveness of prenatal yoga on delivery outcomes: A meta-analysis. Complement Ther Clin Pract. 2020 May;39:101157.	No outcome of interest.
12.	Sánchez-Polán M, Franco E, Silva-José C, Gil-Ares J, Pérez-Tejero J, Barakat R, Refoyo I. Exercise During Pregnancy and Prenatal Depression: A Systematic Review and Meta-Analysis. Front Physiol. 2021 Jun 28;12:640024.	The meta-analysis does not evaluate yoga individually.
13.	Smith CA, Tuson A, Thornton C, Dahlen HG. The safety and effectiveness of mind body interventions for women with pregnancy induced hypertension and or preeclampsia: A systematic review and meta-analysis. Complement Ther Med. 2020 Aug;52:102469.	Only one study of yoga was included. (for stress)
14.	Stoll K, Swift EM, Fairbrother N, Nethery E, Janssen P. A systematic review of nonpharmacological prenatal interventions for pregnancy-specific anxiety and fear of childbirth. Birth. 2018 Mar;45(1):7–18.	No meta-analysis.
15.	Yu X, Liu Y, Huang Y, Zeng T. The effect of nonpharmacological interventions on the mental health of high-risk pregnant women: A systematic review. Complement Ther Med. 2022 Mar;64:102799.	No meta-analysis.

Supplementary file S3. Meta-analyses that were excluded from included systematic reviews.

Systematic Review	Meta-Analysis	Reason
Corrigan et al. 2022	Page 15 – Quality of life	One clinical trial was only included in the analysis.
Guo et al. 2021	Figure 9 – Anxiety<br>Figure 10 – Depression	Interventions of interest were not separately analyzed from other interventions.
Wang et al. 2022	Figure 3 – 6 Depression	One clinical trial evaluated the effectiveness of yoga on postpartum.

Supplementary file S4. Citation matrix and corrected covered area (CCA) calculation.

	Randomized Controlled Trials		Reviews
	Avin, A.; Khojasteh, F.; Ansari, H. The effect of yoga on anxiety in pregnant women in their first pregnancy of Zahedan city in 2017. Spec. J. Med. Res. Health Sci. 2018, 3, 24–31.	-	Lin et al. 2022
2.	Bapat, R.A.; SonyKumari, H.R.N. The effect of one month yoga intervention on perceived stress and anxiety in pregnant women. J. Women's Health Issues Care 2016, 5, 4.	-	Lin et al. 2022

3.	Beddoe, A.E.; Paul Yang, C.P.; Kennedy, H.P.; Weiss, S.J.; Lee, K.A. The effects of mindfulness-based yoga during pregnancy on maternal psychological and physical distress. J. Obstet. Gynecol. Neonatal Nurs. JOGNN 2009, 38, 310–319.	- Lin et al. 2022
4.	Bershadsky S, et al. The effect of prenatal Hatha yoga on affect, cortisol and depressive symptoms. Complement Ther Clin Pract. 2014;20(2):106–113.	- Corrigan et al. 2022<br>- Zhu et al. 2021
5.	Bhartia N, et al. Effects of Antenatal Yoga on Maternal Stress and Clinical Outcomes in North Indian Women: A Randomised Controlled Trial. JIACM. 2019;20(1):10–14.	- Corrigan et al. 2022
6.	Davis, K.J., 2014. The feasibility of yoga in the treatment of antenatal depression and anxiety: a pilot study. ProQuest Inf. Learn. 74, No–Specified.	- Zhu et al. 2021
7.	Davis K, et al. A randomized controlled trial of yoga for pregnant women with symptoms of depression and anxiety. Complement Ther Clin Pract. 2015;21(3):166–172.	- Corrigan et al. 2022<br>- Ng et al. 2019<br>- Ningrum et al. 2019<br>- Smith et al. 2019<br>- Wang et al. 2022
8.	Deshpande CS, et al. Yoga for high-risk pregnancy: a randomized controlled trial. Ann Med Health Sci Res. 2013;3(3):341–344.	- Corrigan et al. 2022<br>- Guo et al. 2021
9.	Field T, et al. Yoga and massage therapy reduce prenatal depression and prematurity. J Bodyw Mov Ther. 2012;16(2):204–209.	- Corrigan et al. 2022<br>- Daley et al. 2014<br>- Gong et al. 2015<br>- Lin et al. 2022<br>- Ningrum et al. 2019<br>- Wang et al. 2022<br>- Zhu et al. 2021
10.	Field T, et al. Yoga and social support reduce prenatal depression, anxiety and cortisol. J Bodyw Mov Ther. 2013;17(4):397–403.	- Corrigan et al. 2022<br>- Daley et al. 2014<br>- Gong et al. 2015<br>- Ng et al. 2019<br>- Ningrum et al. 2019<br>- Smith et al. 2019<br>- Wang et al. 2022<br>- Zhu et al. 2021
11.	Field T, et al. Tai chi/yoga reduces prenatal depression, anxiety and sleep disturbances. Complement Ther Clin Pract. 2013;19(1):6–10.	- Daley et al. 2014<br>- Gong et al. 2015<br>- Lin et al. 2022<br>- Ng et al. 2019<br>- Ningrum et al. 2019<br>- Smith et al. 2019<br>- Wang et al. 2022
12.	Gallagher A, et al. Effects of yoga on anxiety and depression for high-risk mothers on hospital bedrest. Complement Ther Clin Pract. 2020;38:101079.	- Corrigan et al. 2022

13.	Hayase M, et al. Effects of maternity yoga on the autonomic nervous system during pregnancy. J Obstet Gynaecol Res. 2018;44(10):1887–1895.	- Corrigan et al. 2022
14.	Kusaka, M.; Matsuzaki, M.; Shiraishi, M.; Haruna, M. Immediate stress reduction effects of yoga during pregnancy: One group pre-post test. Women Birth J. Aust. Coll. Midwives 2016, 29, e82–e88.	- Lin et al. 2022
15.	Kundarti F, et al. Effect of prenatal yoga on anxiety, cortisol and sleep quality. J Crit Rev. 2020;7(13):655–661.	- Corrigan et al. 2022
16.	Kundarti, F.I.; Titisari, I.; Sepdianto, T.C.; Karnasih, I.G.A.; Sugijati, S. The effect of prenatal yoga on anxiety, cortisol and sleep quality. Int. J. Pharm. Res. 2020, 12, 2268–2276.	- Lin et al. 2022
17.	Mitchell J, et al. Yoga reduces prenatal depression symptoms. Psychology. 2012;3(9A):782–6.	- Corrigan et al. 2022<br>- Daley et al. 2014<br>- Gong et al. 2015<br>- Lin et al. 2022<br>- Zhu et al. 2021
18.	Mohyadin E, et al. The effect of practicing yoga during pregnancy on labor stages length, anxiety and pain: a randomized controlled trial. J Complement Integr Med. 2021;18(2):413–417.	- Corrigan et al. 2022
19.	Muzik, M.; Hamilton, S.E.; Lisa Rosenblum, K.; Waxler, E.; Hadi, Z. Mindfulness yoga during pregnancy for psychiatrically at-risk women: Preliminary results from a pilot feasibility study. Complement. Ther. Clin. Pract. 2012, 18, 235–240.	- Lin et al. 2022
20.	Newham JJ, et al. Effects of antenatal yoga on maternal anxiety and depression: A randomized controlled trial. Depress Anxiety. 2014;31(8):631–640.	- Corrigan et al. 2022<br>- Gong et al. 2015<br>- Ng et al. 2019<br>- Wang et al. 2022<br>- Zhu et al. 2021
21.	Rong L, et al. Efficacy of yoga on physiological and psychological discomforts and delivery outcomes in Chinese primiparas. Complement Ther Clin Pract. 2021;44:101434.	- Corrigan et al. 2022
22.	Ruqaiyah NA, et al. Anxiety Level Assessment to Primigravidae Women (28–40 week) with Hamilton Anxiety Rating Scale (HARS). Method. Indian J Forensic Med Toxicol. 2020;14(4):3301–3306.	- Corrigan et al. 2022
23.	Satyapriya M, et al. Effect of integrated yoga on stress and heart rate variability in pregnant women. Int J Gynecol Obstet. 2009;104(3):218–222.	- Corrigan et al. 2022<br>- Guo et al. 2021
24.	Satyapriya M, et al. Effect of integrated yoga on anxiety, depression & wellbeing in normal pregnancy. Complement Ther Clin Pract. 2013;19(4):230–236.	- Corrigan et al. 2022<br>- Gong et al. 2015<br>- Ng et al. 2019<br>- Ningrum et al. 2019<br>- Wang et al. 2022<br>- Zhu et al. 2021
25.	Shahtaheri, E.; Hosseinian, S.; Ebrahimi, M.; Jalilabadi, Z.; Mirderikvand, F. The impact of stress reduction program based on mindfulness and conscious yoga on depression, perceived stress and quality of life in pregnant women. Acta Med. Mediterr. 2016, 32, 2047–2050.	- Lin et al. 2022

26.	Shu, L.; Xi, M.; Wu, C.; Zhao, W.; Tan, C.; Chen, D. Effects of mindfulness yoga training on sleeping, anxiety and childbirth fear among pregnant women. Chin. Nurs. Manag. 2018, 18, 1422–1427.	-	Lin et al. 2022
27.	Shu, L., Tan, C., Wu, C., Xi, M., Tan, H., Zhao, W., Qu, W., 2019. Study on the intervention effect of group mindfulness yoga on postpartum depression of secondchild pregnant women. Chin. Gen. Pract. 22, 2739–2743	-	Zhu et al. 2021
28.	Uebelacker LA, et al. A pilot randomized controlled trial comparing prenatal yoga to perinatal health education for antenatal depression. Arch Women's Ment Health. 2016;19(3):543–547.	-<br>-<br>-<br>-<br>-	Corrigan et al. 2022<br>Lin et al. 2022<br>Ng et al. 2019<br>Smith et al. 2019<br>Zhu et al. 2021
29.	Vieten C, Astin J. Effects of a mindfulness-based intervention during pregnancy on prenatal stress and mood: results of a pilot study. Arch Womens Ment Health 2008;11(1):67–74.	-	Daley et al. 2014
30.	Yulianti I, et al. The Effect of Prenatal Yoga on Anxiety and Depression in Kudus. Central Java. J Matern Child Health. 2018;3(2):100–104.	-<br>-<br>-	Corrigan et al. 2022<br>Lin et al. 2022<br>Ningrum et al. 2019
31.	Yuvarani G, et al. A study to compare the effects of aerobic exercises and yoga on depression and maternal anxiety orienting among primiparous women. Biomedicine. 2020;40(3):395–8.	-	Corrigan et al. 2022
32.	Zhang, H., Ning, H., Ma, Li, 2016. Effects of prenatal yoga practice on depression and anxiety in pregnant women. J. Aerosp. Med. 27, 1398–1399.	-	Zhu et al. 2021

$$CCA = \frac{N-r}{rc-r} = \frac{77-32}{320-32} = \frac{45}{288} = 0.16 = 16\%$$

Note: n is the number of included publications (including double counting) in the available evidence synthesis (this is the sum of the ticked boxes in the citation matrix); where *r* is the number of rows (number of index publications), and c is the number of columns (number of reviews).
